# Reduced Glycemic Variability in Diazoxide-Responsive Children with Congenital Hyperinsulinism Using Supplemental Omega-3-Polyunsaturated Fatty Acids; A Pilot Trial with MaxEPA^R^

**DOI:** 10.3389/fendo.2014.00031

**Published:** 2014-03-12

**Authors:** Mars Skae, Hima Bindu Avatapalle, Indraneel Banerjee, Lindsey Rigby, Andy Vail, Peter Foster, Christiana Charalambous, Louise Bowden, Raja Padidela, Leena Patel, Sarah Ehtisham, Karen E. Cosgrove, Mark J. Dunne, Peter E. Clayton

**Affiliations:** ^1^Department of Paediatric Endocrinology, Manchester Academic Health Science Centre, Royal Manchester Children’s Hospital, Central Manchester University Hospitals NHS Foundation Trust, Manchester, UK; ^2^Institute of Human Development, Faculty of Medical and Human Sciences, University of Manchester, Manchester, UK; ^3^Institute of Population Health, Faculty of Medical and Human Sciences, University of Manchester, Manchester, UK; ^4^School of Mathematics, University of Manchester, Manchester, UK; ^5^Faculty of Life Sciences, University of Manchester, Manchester, UK

**Keywords:** congenital hyperinsulinism, hypoglycemia, clinical trial, omega-3-polyunsaturated fatty acids, diazoxide

## Abstract

**Objective:** Congenital hyperinsulinism (CHI) is a rare condition of hypoglycemia where therapeutic options are limited and often complicated by side-effects. Omega-3-polyunsaturated fatty acids (PUFA), which can suppress cardiac myocyte electrical activity, may also reduce ion channel activity in insulin-secreting cells. PUFA supplements in combination with standard medical treatment may improve glucose profile and may reduce glycemic variability in diazoxide-responsive CHI.

**Design:** Open label pilot trial with MaxEPA^R^ liquid (eicosapentaenoic and docosahexaenoic acid) PUFA (3 ml/day for 21 days) in diazoxide-responsive CHI patients (https://eudract.ema.europa.eu/, EudraCT number 201100363333).

**Methods:** Glucose levels were monitored pre-treatment, end of treatment, and at follow-up by subcutaneous continuous glucose monitoring systems (CGMS) in 13 patients (7 girls) who received PUFA. Outcome measures were an improved glucose profile, reduced glycemic variability quantified by a reduction in the frequency of glucose levels <4 and >10 mmol/l, and safety of PUFA. All children were analyzed either as intention to treat (*n* = 13) or as per protocol (*n* = 7).

**Results:** Mean (%) CGMS glucose levels increased by 0.1 mmol/l (2%) in intention to treat and by 0.4 mmol/l (8%) in per protocol analysis (*n* = 7). The frequency of CGMS <4 mmol/l was significantly less at the end of treatment than in the pre-treatment period [556 (7%) vs. 749 (10%)]. Similarly, the frequency of CGMS >10 mmol/l, was also less at the end of treatment [27 (0.3%) vs. 49 (0.7%)]. Except for one child with increased LDL cholesterol, all safety parameters were normal.

**Conclusion:** MaxEPA^R^ was safe and reduced glycemic variability, but did not increase glucose profiles significantly in diazoxide-responsive CHI. The supplemental value of PUFA should be evaluated in a comprehensive clinical trial.

## Background

Congenital hyperinsulinism (CHI) is the commonest cause of severe hypoglycemia in children (1, 2) and is associated with adverse neurodevelopmental morbidity in about a third of cases (3). The treatment of hypoglycemia in CHI is complex and challenging. Medical therapy is limited to diazoxide as first line and octreotide as second line choices for the long-term treatment of CHI, with intravenous glucagon use limited to early intervention in the majority of patients (1, 4, 5). However, both diazoxide and octreotide are complicated by inefficacy and adverse effects (4–10). Near total or subtotal pancreatectomy is an option in children who do not respond to medical management. However, the incidence of iatrogenic diabetes, requiring insulin treatment remains high in those who undergo a near total procedure (11). While novel treatment options such as glucagon like peptide 1 antagonists have been tested in older children with CHI, routine clinical application is not yet advocated, pending further studies (12). Hence, there is a significant need to develop further treatment options in the medical management of CHI (13).

Purified fish oils, as food supplements or prescribed formulations containing the polyunsaturated fatty acids (PUFA), eicosapentaenoic acid (EPA) and docosahexaenoic acid (DHA), have been used in the prevention of cardiac arrhythmias following acute myocardial injury (14–16). The mechanism of action of these agents includes a suppression of electrical activity in cardiac myocytes mediated by the inhibition of voltage-gated Na^+^ and Ca^2+^ ion channels, which will prolong the relative refractory periods of the cardiac action potential and prevent cytosolic calcium overload during ischemic events (17, 18). CHI involves the generation of inappropriate electrical activity in β-cells. This occurs either as a direct consequence of mutations in the ATP-sensitive potassium (K_ATP_) channel genes or as a result of metabolopathies leading to enforced K_ATP_ channel closure (1, 4, 19). Diazoxide reduces transmembrane potentials in β-cells by binding to and modulating K_ATP_ channel status (20), and this effect is utilized in the medical therapy of CHI (1). However, diazoxide binding to K_ATP_ channels is incomplete (21) and can be associated with some variability in glucose levels, particularly with illness episodes and variation in nutrition. It is recognized that conductance across K_ATP_ channels in animal models is subject to slow oscillation (22), which may explain the observed variation in diazoxide treatment response. Further, response to diazoxide therapy is not predictably dose dependent; there are case reports of paradoxical hypoglycemia with diazoxide treatment in children with CHI (23). Therefore, while patients on diazoxide are responsive to treatment by demonstrating glycemic stability to a safety fast (1, 5), minor glucose fluxes, both high and low are expected, although occurring infrequently. Although hyperglycemia is not a common cause for concern in the routine management of CHI, glucose intolerance in normal individuals following partial insulin inhibition with diazoxide has been recognized (24). It is likely that children with CHI also experience occasional hyperglycemia in response to treatment with diazoxide, particularly during intercurrent illnesses. Further, glucose-induced electrical activity in β-cells is likely to be modulated by other cellular channel mechanisms, although specific models are yet to be determined (25, 26). We hypothesized that PUFA may exert additional suppression of electrical activity in insulin-secreting cells and that this may lead to a further reduction in inappropriate insulin secretion in patients with CHI. PUFA has an established satisfactory safety profile in adults and children (27, 28) and its status as a food supplement makes fish oil-derived PUFA a potentially attractive treatment adjunct in children with CHI. Supplementing diazoxide with PUFA in the treatment regimen of CHI patients may offer tighter glycemic control and therefore potentially better long-term outcomes. Here, we have reported for the first time, the supplemental use of MaxEPA^R^ on glycemic profile and glycemic variability in diazoxide-responsive CHI.

## Materials and Methods

We aimed to investigate if the addition of PUFA improved glycemic profile and reduced glycemic variability in children with CHI. Only children who were responsive to diazoxide were chosen for recruitment, this being a pilot trial of an experimental food supplement with no previous treatment experience of PUFA in CHI patients. Children between the ages of 6 months and 11 years, with confirmed persistent CHI, and treated with diazoxide with satisfactory glycemic stability were invited to participate in the trial of purified PUFA. Diazoxide responsiveness was defined as achieving euglycemia to a dose of <15 mg/kg/day, satisfactory tolerance to a safety fast at discharge from hospital (1) using age appropriate fasting periods, and >90% of home blood glucose measurements within a range of 4–6 mmol/l, over a 1-month period, with no symptoms or signs of neuroglycopenia. The presence or absence of a known CHI-related gene mutation did not preclude participation in the trial, as there is no currently available information regarding glycemic responsiveness to PUFA supplementation in children with and without CHI mutations. The trial was ethically approved with an ethics committee reference number 11/NW/0549 and European Union trial number 2011-003633-33. Informed consent was obtained from parents of children with CHI.

The primary outcome of the study was to demonstrate an increase in glycemic profile with PUFA supplementation. This being a pilot study, with no previous data, the increment in glucose to achieve a satisfactory outcome following PUFA treatment was not specified. However, as an estimate, the study protocol proposed a sample number of 15 children to detect a mean glucose increment of 1 mmol/l with 85% power, assuming a group standard deviation of 1 mmol/l. The study also evaluated glycemic variability, quantified by the risk of developing hypoglycemia before and after PUFA supplementation. While hypoglycemia was defined as glucose <2.6 mmol/l, measurements with glucose levels <4 mmol/l (i.e., below the normal glucose range of 4–6 mmol/l), commensurate with trends to develop hypoglycemia detected by *in vivo* sampling using continuous glucose monitoring (29, 30), were also analyzed. Similarly, hyperglycemia was defined as glucose >10 mmol/l, for the study cohort (31), with risk of hyperglycemia being defined as glucose >6 mmol/l.

**Figure 1:**
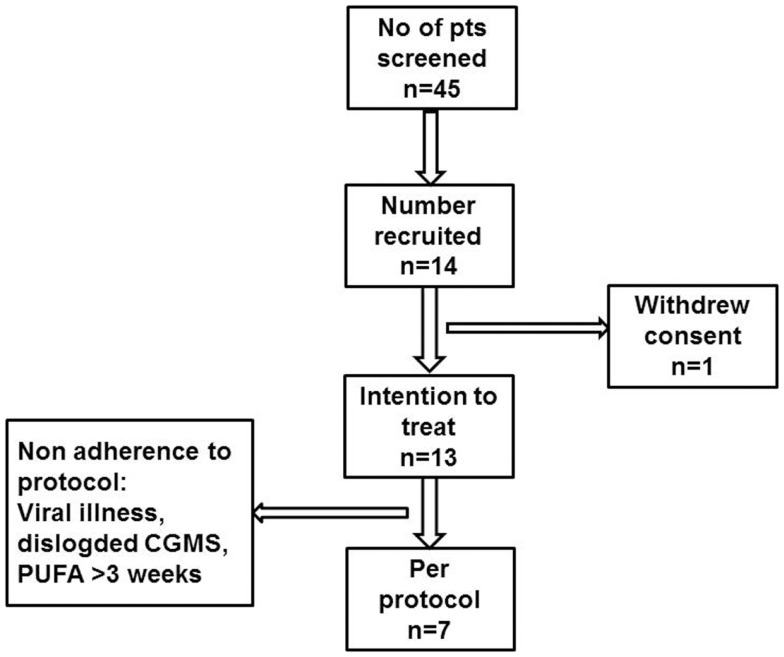
**Participant flow diagram describing patient recruitment to the PUFA pilot trial in children with CHI**.

Children already established on diazoxide treatment (5–12 mg/kg/day) were invited to participate in the trial (Figure 1). These children had satisfactory glycemic stability defined by tolerance to an age appropriate fast, with no reported episodes of symptomatic neuroglycopenia in the month preceding recruitment to the trial. Further, their frequency of relatively low blood glucose levels (<4.0 mmol/l) on home blood glucose monitoring was less than once a week. Children on diazoxide who were less stable than the above definition were excluded from participation to the study, as they required dose adjustments and therefore their recruitment to a pilot study would be inappropriate. A total daily diazoxide dose <6 mg/kg/day was considered to be a low dose, while higher values were considered as relatively high dosage. Gastrostomy tube feeding was permitted at entry, provided that tube feeding was initiated for feed intolerance, not for achieving glycemic stability and that feeds were administered as boluses, at intervals of at least 4 h. The use of cephalosporins, warfarin, or other anticoagulation was not permitted due to anticipated adverse effects on coagulation and such treatment would lead to withdrawal from the trial.

Purified fish oil (MaxEPA^R^ liquid, Seven Seas Limited, Kingston-upon-Hull, UK; Marketing authorization number: PL01932/0003, 1.1 ml = 1 g; each gram containing 170 mg EPA and 115 mg DHA) was administered in a dose of 3 ml/day (EPA 459 mg, DHA 310 mg/day) for a total of 21 days. The daily dose was calculated to be similar to previous drug trial doses in adults (14) and to those used in children (28). As this was a pilot trial, and not a dose finding study, PUFA dose was not specified for age or body weight. Investigation, monitoring, and treatment of patients were performed as specified in Table 1. Parents were required to keep a daily food, activity logbook, and medication compliance diary for each patient with twice daily blood glucose measurements before breakfast and dinner, using the following glucometers: GlucoMen LxPlus (Menarini Diagnostics, UK), OneTouch^®^Ultra^®^2 (Lifescan, UK), and Accu-Chek^®^ Aviva (Roche, UK). All glucometers were calibrated as per manufacturers’ recommendation, prior to the start of the trial. Documented adherence to the trial conditions, treatment dose, and the amount of purified fish oil-derived PUFA consumed was monitored during each study visit to ensure compliance. Parents were also required to return empty medication bottles at the end of treatment period.

**Table 1:** **Investigation, monitoring, and treatment protocol for patients with CHI recruited to the trial, showing time, time periods, and procedures in columns (BP, blood pressure)**.

Day	Period	Procedures
Day 1	Pre-treatment	Consent form; baseline observations (heart rate and BP); 48 h subcutaneous continuous glucose monitoring; baseline blood investigations (fasting blood glucose, insulin, lipids, liver function tests); education of parents regarding administration of fish oil
Day 3	On-treatment	Start 3 week trial of fish oil treatment
Day 10	On-treatment	Baseline observations (heart rate and BP); blood investigations (fasting blood glucose, insulin, lipids, liver function tests); monitoring of log book and diary
Day 23	End of treatment	Baseline observations (heart rate and BP); repeat 48 h continuous glucose monitoring; blood investigations (fasting blood glucose, insulin, lipids, liver function tests); monitoring of log book activity
Day 44	Follow-up	Baseline observations (heart rate and BP); repeat 48 h continuous glucose monitoring; blood investigations (fasting blood glucose, insulin, lipids, liver function tests); advice to continue diazoxide; monitoring of log book activity

*For analysis, the following time periods represented the chronology of trial treatment: pre-treatment, on-treatment, end of treatment, and follow-up*.

Continuous glucose monitoring systems (CGMS) was used to monitor subcutaneous glucose at frequent intervals to determine glycemic control. CGMS glucose is recognized as a useful tool to represent and compare glycemic trends over time (32, 33) and was therefore utilized as the primary method to assess glycemic control in relation to PUFA supplementation. In addition, home blood glucose monitoring, although infrequent in measurement (with a minimal frequency of twice daily) was also utilized as a marker of glycemic control. Blood glucose monitoring was performed four times daily for the duration of CGMS monitoring. Blood glucose monitoring frequency at other times was continued as per the child’s usual home monitoring frequency, although a twice daily frequency was set as a minimum requirement. Frequent laboratory blood glucose levels were not measured owing to the practical and logistic difficulties of venous sampling in children. The iPro™ 2 (Medtronic Limited, Watford, UK) CGMS device was inserted subcutaneously at specified time periods before treatment, at the end of treatment, and in the follow-up period to provide trends in glycemic status. CGMS readings, recorded every 5 min, were downloaded for analysis.

Adverse events were monitored at each visit to the treatment center. Provisions were made to monitor serious adverse reactions (SAR), whether expected SAR or suspected unexpected serious adverse reactions (SUSAR). Criteria to stop the trial included new safety data, or concerns from safety data (number and nature of SUSARs) or evidence from other studies. Data derived from this study was analyzed by SPSS 20.0 (IBM©United States, 2011) using paired *t*-tests for comparison of data between pre-treatment and end of treatment periods. The statistical software R (R Foundation for Statistical Computing, Austria, 2013) was used for graphical representation and for construction of smoothed trajectories of CGMS glucose profiles in the pre-treatment and end of treatment periods (34).

## Results

Fourteen sets of parents and children with CHI were approached to participate in the trial (Figure 1). Although parents of all 14 children provided consent, 1 child withdrew assent and was excluded from the study. Thirteen children (patient #1–13) (six boys, seven girls) were recruited to the trial and received study drug, therefore constituting the intention to treat group. At entry to the trial, the median (range) age of the children was 5.9 (1.0; 11.9) years. The median (range) age at diagnosis of hypoglycemia was 1 (1; 750) day, with insulin levels of 9.0 (3.0; 20.0) mU/l when diagnosed with CHI. Early neonatal diagnosis (age <7 days) was noted in 8 (61%). No mutations were found in *ABCC8*/*KCNJ11* in 10 (77%) children, while in 3 children, the following mutations were found: paternally heterozygous *KCNJ11* (*n* = 1, patient #13), paternally heterozygous *GCK* (*n* = 1, patient #10), and *HADH* (*n* = 1, patient #4). Positron emission and computed tomography (PET–CT) scans using 6-l-^18^F-fluorodihydroxyphenylalanine (^18^F DOPA) were performed in the child with a heterozygous *KCNJ11* mutation and in eight other children with no mutations, as per local clinical protocol (13). All scans suggested non-focal etiology. All recruited children were treated with diazoxide in a dose of 8.3 (5.0; 12.0) mg/kg/day at the time of recruitment to the study, and were responsive to treatment with no unacceptable adverse effects to consider alternative medical therapy. All recruited children were managed at home with visits to the trial center as per protocol.

Suspected unexpected serious adverse reactions were not reported in any of the 13 children recruited to the trial. However, in six children, protocol deviation was recorded in the following aspects: duration of PUFA treatment less than (patient #3) or beyond (patient #5, #10) 3 weeks, monitoring stopped due to concomitant infection (patient #7), home blood glucose not measured (patient #2), and CGMS duration <48 h (patient #2, #13). Therefore, pooled data from a total of seven children (three boys, four girls) of median (range) 4.2 (1.4; 7.9) years were further analyzed as per protocol.

### Glycemic status in response to PUFA supplements

**Figure 2:**
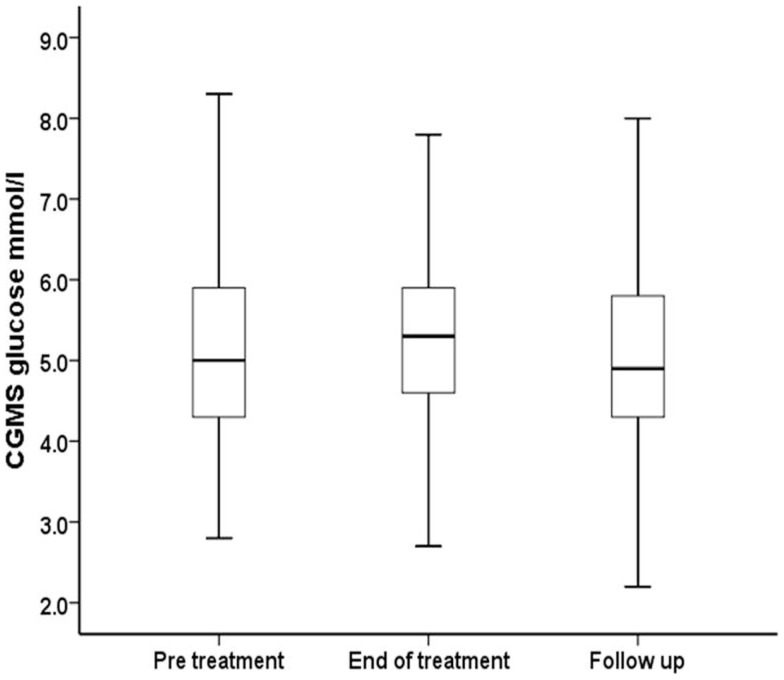
**Median, interquartile range, and ±2 SD values for CGMS glucose (millimoles per liter) have been summarized for all patients receiving PUFA supplements in intention to treat analysis**.

**Figure 3:**
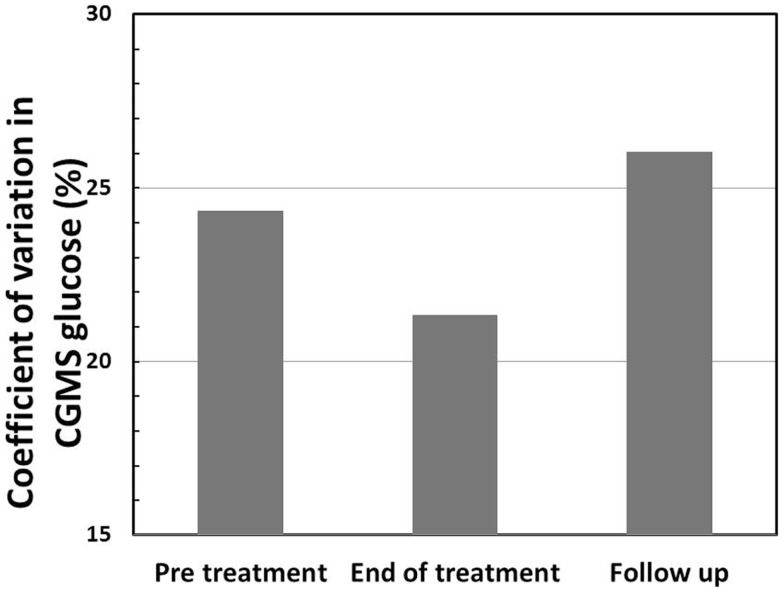
**Coefficient of variation (CV) (%) in CGMS glucose levels shows a reduction at the end of treatment in intention to treat analysis, indicating reduced variability in glucose profiles following treatment with PUFA**.

**Figure 4:**
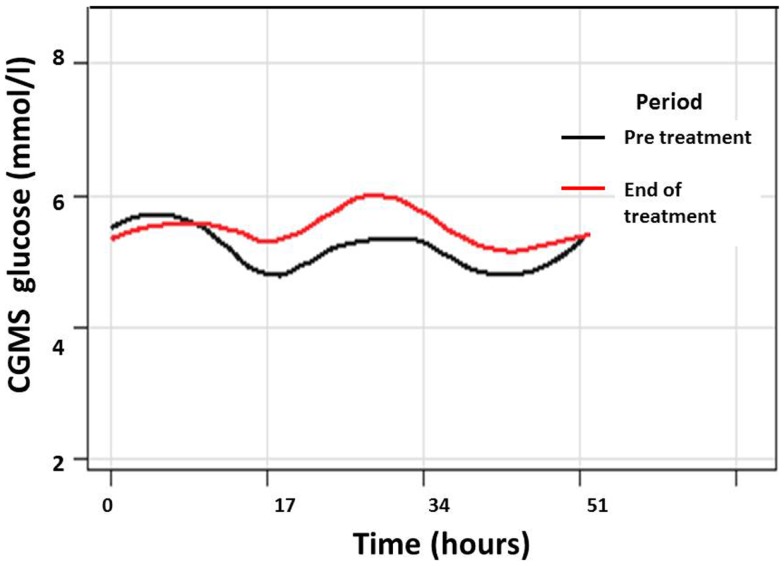
**Composite graph of smoothed trajectories of CGMS glucose profiles for all patients analyzed per protocol (*n* = 7) in the pre-treatment (black line) and end of treatment (red line) periods show overall improvement in glycemic control following PUFA treatment**. Individual glucose values have not been shown for clarity.

**Figure 5:**
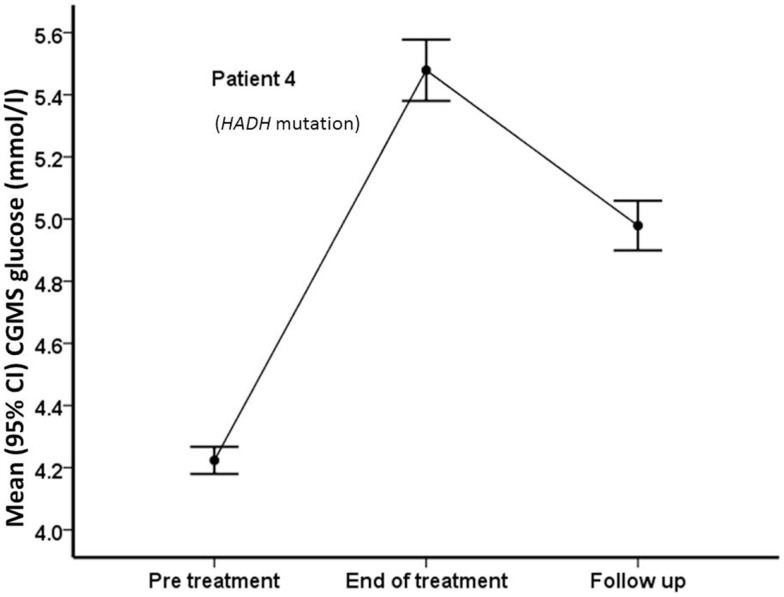
**Mean (95% confidence intervals) CGMS levels at pre-treatment, end of treatment, and follow-up time periods in patient #4, who carries a *HADH* mutation**. Following treatment with PUFA, the patient’s CGMS glucose increased significantly, with reduction after treatment was discontinued.

Continuous glucose monitoring system glucose levels, recorded for all patents in intention to treat and per protocol analyses, have been summarized in Table 2A. Overall, there was a small (mean increase 0.10 mmol/l, 1.9%, *p* < 0.001) clinically insignificant increase in CGMS glucose levels at the end of treatment in intention to treat analysis. The increment was greater (mean increase 0.40 mmol/l, 7.5%, *p* < 0.001) in per protocol analysis. Individual CGMS responses for patients in intention to treat analysis were variable, as shown by the spread of glucose levels around the median value in Figure 2. Although CGMS glucose levels remained variable at all three treatment periods, the variability was less at the end of treatment as shown by the lower coefficient of variation (representing a normalized measure of dispersion) in Figure 3. The amalgamated, smoothed trajectories of CGMS glucose profiles showed an overall improvement in per protocol analysis (Figure 4). An individual example of a positive response to PUFA supplement is shown in Figure 5. Pre-meal blood glucose levels, summarized in Table 2B, were unchanged in intention to treat (mean increase 0.1 mmol/l, 1.8%, *p* = 0.59) and increased minimally in per protocol analyses (mean increase 0.38 mmol/l, 7.0%, *p* = 0.13).

**Table 2:** **(A) Mean (SD) pre-meal CGMS glucose levels (millimoles per liter) have been shown for each time period: pre-treatment, end of treatment, and follow-up, both in an intention to treat analysis (*n* = 13) and per protocol analysis (*n* = 7). (B) Mean (SD) pre-meal blood glucose levels (millimoles per liter) have been shown for each time period: pre-treatment, on-treatment, end of treatment, and follow-up, both in an intention to treat analysis (*n* = 13) and per protocol analysis (*n* = 7)**.

Treatment period	Intention to treat (*n* = 13)		Per protocol (*n* = 7)	
	Number of readings	Mean (SD)	Number of readings	Mean (SD)
**(A) CGMS glucose levels**				
Pre-treatment	7375	5.26 (1.28)	3930	5.29 (1.18)
End of treatment	7984	5.36 (1.14)	4307	5.69 (1.13)
Follow-up	8465	5.19 (1.35)	4219	5.45 (1.50)
**(B) Blood glucose levels**				
Pre-treatment	140	5.44 (1.68)	78	5.41 (1.41)
On-treatment	862	5.29 (1.48)	422	5.49 (1.65)
End of treatment	144	5.54 (1.54)	79	5.79 (1.69)
Follow-up	693	5.35 (1.56)	381	5.29 (1.36)

*CGMS glucose was not measured in the on-treatment period, unlike blood glucose levels*.

At the time of PUFA supplementation, six children were treated with low dose diazoxide (dose <6 mg/kg/day). The effect of low dose diazoxide on CGMS glucose levels following PUFA supplementation was analyzed by a two-way ANOVA model (adjusted *R*^2^ = 0.03, *p* < 0.001). In addition to the effects of PUFA on glucose, low dose diazoxide was independently associated with a higher mean (SD) glucose level than high dose diazoxide [5.4 (1.2) vs. 5.2 (1.0), *p* < 0.001]. The interaction of PUFA and low dose diazoxide was also associated with a higher glucose level (*p* < 0.001), suggesting that PUFA supplementation in children on low dose diazoxide may be more beneficial in improving the glycemic profile than those receiving relatively higher dosage.

### Variability of glycemic status in response to PUFA supplements

**Figure 6:**
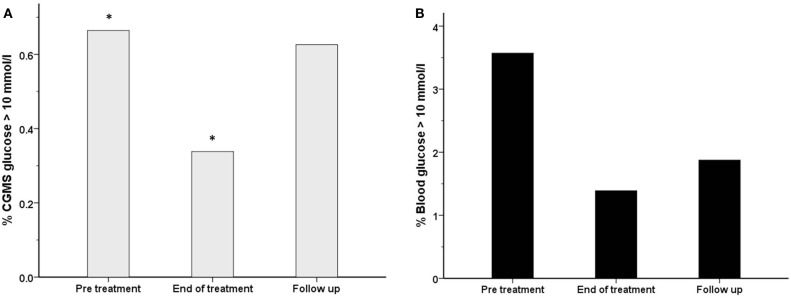
**The frequency (%) of measurements with a glucose level >10 mmol/l has been shown for each treatment period for both blood glucose (black filled bars) (B) and CGMS glucose (gray bars) (A) in intention to treat analysis**. There was a reduction in the frequency of blood glucose >10 mmol/l from 3.6% (pre-treatment) to 1.4% (end of treatment), *p* = 0.27. Similarly, the frequency of CGMS glucose >10 mmol/l was reduced from 0.7 to 0.3%, *p = 0.004.

The frequency of CGMS glucose >10 mmol/l, indicating hyperglycemia, was lower at end of treatment compared to the pre-treatment period, in intention to treat analysis [27 (0.3%) vs. 49 (0.7%), *p* = 0.004] (Figure 6A). However similar results were not replicated in per protocol analysis, as hyperglycemia was not observed in the pre-treatment period. Similarly, the frequency of blood glucose >10 mmol/l was also less at the end of treatment than at pre-treatment (Figure 6B), although the difference was not statistically significant [2 (1.4%) vs. 5 (3.6%), *p* = 0.27], a reflection of the relatively small sample size. The frequency of CGMS glucose >6 mmol/l reflecting the risk of hyperglycemia was also tested, but was similar between pre-treatment and end of treatment periods.

**Figure 7:**
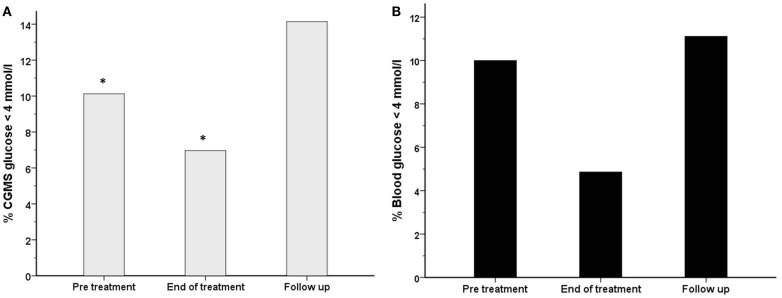
**The frequency (%) of measurements with a glucose level <4 mmol/l has been shown for each treatment period for both blood glucose (black filled bars) (B) and CGMS glucose (gray bars) (A) in intention to treat analysis**. There was a reduction in the frequency of blood glucose <4 mmol/l from 10% (pre-treatment) to 4.9% (end of treatment), *p* = 0.09. Similarly, the frequency of CGMS glucose <4 mmol/l was reduced from 10.1 to 7%, *p < 0.001.

The frequency of CGMS glucose <4 mmol/l was significantly less at the end of treatment than in the pre-treatment period [556 (7.0%) vs. 749 (10.1%), *p* < 0.001] in intention to treat analysis (Figure 7A) and also in per protocol analysis [105 (2.4%) vs. 343 (8.7%), *p* < 0.001]. In the follow-up period, the frequency of CGMS glucose <4 mmol/l rebounded to exceed that in the pre-treatment period. Although not significantly different, the frequency of blood glucose <4 mmol/l was also lower at the end of treatment (Figure 7B) [7 (4.9%) vs. 14 (10%), *p* = 0.09]. In contrast, the frequency of CGMS glucose <3.5 mmol/l as an alternative measure of the risk of hypoglycemia was not lower at the end of treatment.

### Adverse effect profile with PUFA supplements

Although blood and CGMS glucose levels >10 mmol/l were recorded at the end of treatment, no child presented with features of persistent hyperglycemia or symptoms to suggest diabetes. Fasting glucose, insulin, and insulin resistance measured by the HOMA-IR index (Table 3) were not different between the start and end of treatment. Similarly, there were no differences in serum alanine transaminase or albumin levels, representing satisfactory liver function. However, serum cholesterol was elevated in one child (pre-treatment 5.1 mmol/l, end of treatment 5.7 mmol/l, follow-up 6.2 mmol/l), which remained elevated (6.7 mmol/l) 3 months after completion of PUFA treatment. In this child, there was a history of familial LDL hypercholesterolemia, although LDL cholesterol levels were within the normal range. It was not possible to convincingly exclude hypercholesterolemia, as a PUFA related side effect and it was therefore reported as an adverse event. Overall, total and LDL fractions of serum cholesterol were elevated after treatment, although remaining within the normal range (Table 3). In contrast, there was no difference in serum triglyceride levels.

**Table 3:** **Median (interquartile range) pre-meal markers of insulin sensitivity, liver function, and lipid profile has been shown for all patients**.

	Pre-treatment	End of treatment	*p* Value for difference
Insulin (mU/l)	1.7 (2.1)	1.6 (3.4)	0.66
Glucose (mmol/l)	4.5 (0.7)	4.5 (0.6)	0.73
HOMA-IR	0.32 (0.53)	0.32 (0.71)	0.89
ALT (U/l)	17.0 (10.0)	18.0 (17.0)	0.45
Albumin (g/dl)	42.0 (4.0)	40.0 (4.0)	0.96
Cholesterol (mmol/l)	4.1 (1.0)	4.2 (0.7)	0.01*
LDL cholesterol (mmol/l)	2.2 (0.8)	2.7 (0.4)	0.009*
HDL cholesterol (mmol/l)	1.5 (0.5)	1.32 (0.6)	0.55
Triglyceride (mmol/l)	0.6 (0.3)	0.8 (0.4)	0.10

The difference of levels between pre-treatment and end of treatment periods has been assessed by Wilcoxon tests for paired samples. Total and LDL cholesterol levels were increased by a significant (*) margin at the end of treatment, particularly noted in patient #8, although within the normal range.

## Discussion

We report the first pilot trial of the use of supplemental purified PUFA in the treatment of CHI. In this open label, phase 2 trial, although fish oil-derived PUFA supplements given to a group of children with stable glycemic status did not increase CGMS or blood glucose levels by a clinically significant margin, there was significant reduction in the risk of developing both hypoglycemia and hyperglycemia at the end of treatment period, suggesting reduced glycemic variability. There was variability in the glucose response between patients, with some patients responding with a greater increment in glucose levels than others. In one patient with the *HADH* mutation, there was brisk glycemic increment, suggesting that PUFA may be more beneficial in specific forms of CHI, such as those due to lipid disorders. No significant adverse effects of supplementation were noted, except for one patient in whom hypercholesterolemia was observed. It is possible that familial hypercholesterolemia may have been present in this patient, although the possibility of DHA induced LDL hypercholesterolemia cannot be excluded, particularly as total and LDL cholesterol levels were elevated following PUFA treatment (35, 36).

Results from several studies have shown that PUFA increases fasting glucose concentrations in people with type 2 diabetes mellitus and control groups (37–39) and that this might occur by decreasing the sensitivity of pancreatic β-cells to glucose (40). The satisfactory safety profile in several studies in adults and children (14, 27, 28) and its status as a food supplement makes fish oil-derived PUFA an attractive treatment adjunct to achieve greater glycemic stability in children with CHI. In our study, the primary objective of increased glycemic response was not achieved and the extent of glucose increment observed was lower than hypothesized in the trial protocol. This may have been due to several factors, including small sample size, low dose and duration of PUFA, age differences, and variability in diazoxide dose. However, a small but positive upward trend was noted, particularly in longitudinal analysis. The magnitude of glucose response was greater in those on lower doses of diazoxide, suggesting a differential PUFA response with diazoxide sensitivity. While this finding may suggest greater benefit in a select group of patients, the results should be tested in a larger cohort to minimize small sample bias and variability within the cohort.

It is possible that PUFA does not induce hyperglycemia in the basal state while glycemic stability is already achieved, but reduces the risk of developing hypoglycemia. From our study, it appears that PUFA may reduce large fluxes of glucose, both high and low, thereby achieving a more physiological profile in diazoxide treated patients with CHI. The trend to a decrease in the frequency of glucose <4 mmol/l was similar between CGMS and blood glucose measurements and consistent for intention to treat and per protocol analyses. In contrast, the reduction in frequency of hyperglycemia was not consistent between intention to treat and per protocol analyses, which may suggest a greater effect of PUFA in reducing the risk of hypoglycemia, but not hyperglycemia. The frequency of hyperglycemia was also dissimilar between blood and CGMS measurements. This may be due to CGMS measurements being taken continuously, while the majority of blood glucose levels correlated to mealtimes only. The differences in frequency of measurement between the two methods and the possibility that CGMS may be less sensitive in the detection of hypoglycemia than blood glucose monitoring (41) may also explain the variation. For a future trial, a prolonged duration of CGMS for each period related to treatment may be useful.

We did not address the mechanisms of action of the purified omega-3 acid ethyl esters on isolated islets from CHI patients in this study. However, since PUFA leads to a shift in voltage dependence of inactivation in the hyperpolarizing direction for voltage-gated Ca^2+^ and Na^+^ channels in cardiac myocytes (42, 43), a similar shift in the voltage dependence of calcium channel inactivation in pancreatic β-cells would be expected to reduce hypoglycemia by attenuating Ca^2+^ influx-dependent insulin secretion. In conjunction with diazoxide, PUFA may potentiate membrane stabilization in β-cells, leading to an overall decrease in glycemic variability; however, such a mechanism remains unproven and therefore speculative.

As this was a pilot study in a rare condition, the number of children with CHI recruited to the trial were relatively few. Not all children in the trial adhered strictly to the protocol. We have therefore adjusted our results to include both intention to treat and per protocol analyses. The study was designed to measure subcutaneous glucose levels before treatment and at the end of treatment, but not during treatment. In the treatment phase, only blood glucose levels by home monitoring devices were recorded. As an alternative, CGMS monitoring could have been utilized; however, the frequent multiple insertion of subcutaneous needle devices was deemed intrusive and impractical and therefore not incorporated in the study design.

We did not standardize the meter used for home glucose monitoring, which may have introduced variability. It is widely recognized that home blood glucose monitoring devices can be technically unreliable, particularly at low values (44). Therefore, our results would have been more accurate had we monitored more frequently and used a more robust method for measuring blood glucose, such as a reliable point of care testing or a formal laboratory analysis. The latter would, however, have required prolonged hospital admission and generated undue distress in individuals and introduced a bias in the results. In contrast to blood glucose monitoring, CGMS readings were more numerous and therefore captured continuous trends over longer periods of time.

Our data show that PUFA brings about a reduction in the tendency to develop hypoglycemia and this is an important observation in children who are already receiving moderately large doses of diazoxide. In this situation, the addition of PUFA may bring about greater glycemic stability, while perhaps allowing a diazoxide dose reduction to minimize adverse effects of this drug. In those on larger doses of diazoxide with episodic hypoglycemia, PUFA may tighten glycemic control, thereby preventing second line therapy with injectable somatostatin analogs. In our cohort, we have shown that PUFA treatment for patients on low dose diazoxide was associated with higher glucose levels. Therefore, PUFA treatment may aid withdrawal of diazoxide in those on minimal treatment. Whether PUFA supplementation reduces the frequencies of hypo and hyperglycemia at lower thresholds need to be tested in appropriately powered and larger multicentered cohorts. Initial results from the pilot trial involving relatively small number of patients are encouraging and provide a basis on which to conduct a comprehensive clinical trial investigating the utility of PUFA supplements in all patients treated medically for CHI. In such a trial, alternative forms of PUFA, for example, a predominantly EPA containing fatty acid (AMR101) with lesser risk of increasing LDL cholesterol (35, 36, 45) may be considered.

## Conclusion

In our pilot trial, we observed that purified PUFA was safe and reduced glycemic variability, i.e., the risk of both hypoglycemia and hyperglycemia in children with diazoxide-responsive CHI. Although overall glucose levels were not increased significantly, glycemic increment was significant in some children, suggesting positive individual glycemic responses to PUFA. The mechanisms of action associated with reduction in hypo and hyperglycemia remain undetermined, although stabilization of inappropriate ion channel activity in pancreatic β-cells may be involved. Based on our initial results, PUFA may be considered as an additional therapeutic option to tighten glycemic control in diazoxide treated CHI patients. Functional studies and a larger, more comprehensive trial are required to fully investigate the value of PUFA as a treatment adjunct for CHI.

## Author Contributions

Mars Skae, Hima Bindu Avatapalle, Indraneel Banerjee, Lindsey Rigby, Louise Bowden, Raja Padidela, Sarah Ehtisham, Peter E. Clayton were responsible for setting up the trial, data acquisition, recruitment to trial. Mark J. Dunne, Karen E. Cosgrove, Peter E. Clayton were responsible for study conception and design. Mars Skae, Hima Bindu Avatapalle, and Peter E. Clayton coordinated the trial participation. Mars Skae, Hima Bindu Avatapalle, Indraneel Banerjee, Mark J. Dunne, Karen E. Cosgrove, Peter E. Clayton were responsible for manuscript writing, drafting the figures and tables. Andy Vail, Peter Foster, Christiana Charalambous were responsible for study design and statistical analysis. Indraneel Banerjee, Peter E. Clayton, Karen E. Cosgrove were responsible for coordinating the writing of the manuscript. All authors read and approved the manuscript.

## Conflict of Interest Statement

The authors declare that the research was conducted in the absence of any commercial or financial relationships that could be construed as a potential conflict of interest.

## Abbreviations

CGMS, continuous glucose monitoring systems; CHI, congenital hyperinsulinism; DHA, docosahexaenoic acid; EPA, eicosapentaenoic acid; KATP, ATP-sensitive K+ channels; LDL, low density lipoprotein; PUFA, polyunsaturated fatty acids.
